# Applying the multiphase optimization strategy to evaluate the feasibility and effectiveness of an online road safety education intervention for children and parents: a pilot study

**DOI:** 10.1186/s12889-024-19208-z

**Published:** 2024-07-04

**Authors:** Julia Pham, Adrian Buttazzoni, Jason Gilliland

**Affiliations:** 1 Human Environments Analysis Laboratory, Department of Geography, Faculty of Social Sciences, University of Western Ontario, London, ON Canada; 2 Department of Geography and Environment, University of Western Ontario, London, ON Canada; 3 School of Planning, University of Waterloo, Waterloo, ON N2L 3G1 Canada; 4 Children’s Health Research Institute, London, ON Canada; 5 Lawson Health Research Institute, London, ON Canada; 6 Department of Paediatrics, University of Western Ontario, London, ON Canada; 7 Department of Epidemiology & Biostatistics, University of Western Ontario, London, ON Canada; 8 School of Health Studies, University of Western Ontario, London, ON Canada

**Keywords:** Active travel, Feasibility, Health promotion, Implementation science, Intervention, Road safety education

## Abstract

**Background:**

Reports of children’s engagement in active transportation outline low participation rates in many countries despite many associated mental, physical, and social health benefits. One of the main contributors to this phenomenon is a cited lack of education and knowledge among children regarding active travel (AT), specifically road safety. To address this issue, the aim of this study was to evaluate the feasibility and effectiveness of an online road safety education intervention to promote AT among children and their parents.

**Methods:**

Applying the Multiphase Optimization Strategy (MOST) for intervention development, implementation, and evaluation, we designed and assessed a four-module online road safety education intervention with a sample of 57 parent-child dyads using a 2^3^ factorial design featuring both qualitative and quantitative analyses.

**Results:**

Main intervention feasibility findings include positive and critical feedback on the program’s content and design, and moderate participant engagement as reflected by program retention and completion rates. With respect to the preliminary intervention effectiveness on children, a significant improvement in road safety knowledge scores was observed for groups that feature the “wheeling safety and skills” module. Slight improvements in AT knowledge scores across all the intervention groups were observed, but were not of significance. Preliminary intervention effectiveness on select parental AT practices and perceptions saw significant improvements in some groups. Groups that featured the ‘wheeling safety and skills’ module exhibited significantly higher guided choice scores upon completion of the program than those who did not receive this component.

**Conclusion:**

The MOST framework allowed us to design and evaluate the feasibility and preliminary effectiveness of an online road safety education intervention. The developed intervention has demonstrated that it has the potential to improve children’s road safety knowledge and some areas of parental AT practices and perceptions, to which improvements may be attributed to the inclusion of the “wheeling safety and skills” module, suggesting that the targeted focus on cycling skills is a prioritized area. AT programming and practice implications are discussed. Future research is encouraged to refine modules to better reflect the priorities of children and parents and to test these refined components among larger samples.

**Word count:**

9,391 (excludes abstract, tables, figures, abbreviations, and references).

**Supplementary Information:**

The online version contains supplementary material available at 10.1186/s12889-024-19208-z.

## Background

Physical activity (PA) is important for healthy child development and overall health [1, 2]. However, global trends outline continued declines in PA among children with 81% of those between 11 and 17 years not meeting their recommended 60-minutes of daily moderate-to-vigorous PA [3]. Physical inactivity in early life is especially concerning as it has been linked to the development of several chronic health diseases such as diabetes, hypertension, osteoporosis, cardiovascular disease, and obesity [4]. One relatively accessible and inexpensive population-level approach to addressing childhood physical inactivity is the promotion of active travel (AT; i.e., walking/cycling/wheeling), which can not only improve levels of PA but also offers many health benefits [5–7]. In addition to increases in daily moderate-to-vigorous PA [8, 9], regular engagement in AT has been associated with enhanced cardiovascular fitness [10], improved PA habits [11], self-reported mental health [12], and healthier body composition [13]. Despite these potential health benefits, levels of participation in regular or occasional (i.e., ≥ 3 days/week) AT are less than 50% and have remained generally the same or decreased over the last 10–15 years in several countries across the globe [14].

Several different individual, interpersonal, and environmental factors contribute to these observed trends. However, two related and typically cited deterrents to AT among children are poor road safety knowledge (e.g., uncertain how to cross at intersections) and a lack of relevant skills (e.g., cycling skills)—issues that can negatively impact their motivation to engage in AT [15]. Not only does a lack of traffic safety knowledge deter children from participating in AT, but it is one of the key contributors to road traffic injuries, the leading cause of child mortality worldwide [16]. Children are more susceptible to pedestrian injury due to their poor hazard perception and decision making abilities, vital cognitive skills that are expected to improve with increased education and exposure to different traffic-related scenarios [17, 18]. To address these concerns, various road safety education interventions have been developed in recent years with an increasing number utilizing virtual and digital mediums [17, 19, 20]. Digital road safety education interventions might be especially effective regarding the improvement of children’s pedestrian safety behaviour through enhancing their understanding of pedestrian risk factors (e.g., hazard identification) [21, 22] and subsequent abilities to apply these learnings to real-life situations [21, 23]. Although these approaches to AT and injury prevention programming have become more widely adopted with respect to their usage and strategies, more rigorous evaluations of these interventions have been noted [21]. The aim of this paper is to therefore evaluate the feasibility and effectiveness of an online road safety education intervention to promote AT among a sample of children and parents.

### Influences of child AT participation

Individual, interpersonal, community, and environmental factors can all influence a child’s decision and ability to participate in AT. Prominent individual-level factors that factor into AT decision-making processes include age [24, 25], gender [26, 27], personal motivations [28], poor perceived efficacy [29], and access to equipment such as bicycle helmets [30]. Likewise, interpersonal factors related to both peers and family—for example, trip social experience or opportunities to socialize [31, 32], as well as parental education [33] and support [34], respectively—can also impact travel decisions. Parental support for their children’s participation in AT is informed by their perceptions and knowledge of the activity [35, 36]. Such dynamics oftentimes result in parents effectively functioning as “gatekeepers” with respect to their children’s mode of travel decisions to/from various destinations such as school [37]. Importantly, more restrictive attitudes and beliefs among parents can hinder their child’s AT participation. Rules that restrict children to stay within sight of a parent [38], heightened concerns pertaining to neighbourhood safety [39], traffic safety concerns [40], family preferences for school choice, increasing commute distance [41], and the convenience associated with passive travel (i.e., personal vehicles) and other family commuting demands [42] have been documented as barriers. Conversely, supportive attitudes and perceptions can encourage a child’s participation in AT. For instance, positive parental perceptions of the associated social and emotional benefits of AT [12], perceptions of supportive environmental attributes (e.g., presence of parks) [43], greater perceived community social capital or cohesion [28], and support for the potential to commute with peers [44] can promote AT engagement.

AT participation can also be encouraged through improving parental evaluations of their child’s commuting competence [36] and ability to undertake risk assessments during trips [45], which can subsequently lessen worries related to risky pedestrian behaviours [46], while promoting community support for AT [47]. In service of advancing parental support for AT, recent work with parents has recommend that future intervention efforts implement strategies targeting community building, cycling participation, road safety issues [48], and infrastructure safety and accessibility concerns [49]. Social and physical environment factors such as perceptions of neighbourhood safety, population density, and local traffic infrastructure can also impact AT participation [50]. As a determinant of AT, perceptions and education related to the activity cross several of these theoretical boundaries, while intersecting individual, interpersonal, community, and/or environmental considerations.

A child’s ‘willingness to change’—that is, their openness, readiness, and ability to accept alterations to existing behavioural preferences and patterns—can also affect their participation in AT. Willingness to change has been documented as a factor in the analysis of health behaviour change among younger cohorts with respect to physical activity, healthy eating [50], smoking habits [51], and risks related to chronic illness [52]. As it concerns AT participation, as alluded to above, a child’s willingness to change their travel behaviour can be affected by a several factors ranging from individual concerns like poor personal evaluations of one’s cycling skills and interpersonal conflicts like a lack of parental support stemming from overstated safety concerns (e.g., stranger danger), to community-level issues such as a lack of neighbourhood social control (e.g., local crime) [15] and gendered norms related to parental controls and activity suitability that restrict cohorts like younger girls [53]. Indeed, though environment-level influences like infrastructure are often earmarked for interventions to facilitate AT engagement, parental controls, AT abilities (e.g., cycling skills), and personal safety and risk perceptions are other notable pathways through which AT behavioural change has been targeted by initiatives [54]. Consequently, practitioners must consider this range of issues when seeking to positively impact a child’s willingness to change AT behaviours. One notable approach that has been associated with encouraging results regarding both external—e.g., improvements in risk perceptions among drivers [55]—and internal—pedestrian safety behaviours [56]— factors has been AT education strategies.

### Child AT education (perceptions, knowledge, skills)

A child’s AT education encompasses several different factors connected to their decision-making processes, most notably their perceptions, relevant skills, and general knowledge. Common perceived barriers to AT reported among child population have outlined concerns regarding high volumes of street traffic [51], reckless driving [52], and apathy [53]. Conversely, perceptions such as having available group-based commuting arrangements [54], ‘eyes on the street’ from community members [55], and appealing streetscapes [56] among children have been suggested to support their participation in AT. Child-centered research has also conveyed knowledge pertaining to technical considerations like proper bike helmet use [57], awareness of academic [58] or physical and mental health benefits [54], and improved environmental connections and attachments [59]. The development, or lack thereof, of road crossing skills is a central determinant for AT. Road crossing skills in areas adjacent to schools [60], pedestrian risk perceptions [61], attentional and visual searching competence [62], and cycling abilities (e.g., poor turn maneuvers, riding too fast) [63] are particularly important skills that have been connected with AT participation.

#### AT education interventions

To address these myriad education-related factors and promote AT, several precise strategies and broader interventions have been developed and implemented. For instance, Baslington [31] evaluated the effectiveness of a personal, social health and citizenship education (PSHCE) curriculum to support active transportation, an approach which featured multiple class projects where students were introduced to school travel planning (STP) topics and being provided opportunities to keep travel diaries in service of monitoring their travel habits. Likewise, An and Yang [52] conducted a mixed methods evaluation of a multi-component road safety education program for senior-level elementary school students which included strategies aiming to improve knowledge related to road signs, road safety rules, and safe travel practices. Other education-based interventions have sought to implement more specific approaches when promoting AT. Cycling skill development programs featuring varied cycling exercises (e.g., signaling, one- and two-handed steering) [64], cycling knowledge initiatives comprised of simulated scenarios (e.g., demonstrating cycling skills in varied traffic environments) [65], pedestrian street crossing training [66], and traffic observation and safety assessments [67], which have all addressed more specific child AT influences via more targeted strategies. However, recent advances in technology providing for relatively greater and easier access to both broader population-level samples have resulted in an increase in the usage of online and digital mediums with respect to AT intervention development and delivery [20, 68–70]. While there has been a considerable growth in the application of these delivery mediums, more robust intervention designs [71] and evaluations using these approaches have been called for [72].

#### Online and digital road safety education interventions

An emerging approach used to facilitate AT education interventions is the use of online and digital platforms. Increasingly AT programmers have utilized these mediums to employ targeted strategies that address distinct aspects of AT education, notably pedestrian safety and street crossing skills [70–77], road safety awareness [21, 77], and bicycle safety and skills [17, 78, 79].This variety of methodological options is a particularly noteworthy benefit of online and digital programming as interactive multimedia, video clip, video game interventions [19, 79, 80], and virtual reality scenarios [22, 69, 70], as well as combinations of these tools, have all be used to address road safety education topics. While expanding in their implementation, the effectiveness of online education interventions (OEIs) for AT road safety remains uncertain [77, 81]. An important and consistent limitation often hampering this work is a lack of theoretical inputs to guide the design, implementation, and evaluation of such interventions. At present, it appears there is only scattered uses of social cognitive theory [78, 79, 82], despite many studies featuring multi-group designs [17, 74, 83]. Decision-making processes related to a child’s engagement in AT and safe pedestrian behaviour are generally subject to parental controls and practices [22, 35, 36]. Despite parental support being a key component to the development of digital AT and road safety education interventions, several studies lack parental input and/or do not examine parental perceptions or knowledge with respect to intervention effectiveness [17, 73]. Therefore, studies should seek to increase parental involvement in order to better understand children’s strengths and weaknesses related to road safety in furtherance of encouraging children’s willingness to learn and practice safe pedestrian behaviours [22, 23].

### Research aim, questions

Noting the extant methodological concerns pertaining to online and digital intervention approaches, and seeking to design a comprehensive AT education intervention, we applied the multiphase optimization strategy (MOST) [84–86]. A systematic approach to program development and evaluation that is illustrated in Table 1, the MOST is designed to guide the generation of coherent knowledge bases that will subsequently inform the building, optimizing, and evaluating of a particular multicomponent intervention [86]. In sum, the MOST framework is centrally organized according to a three-phase plan: preparation, optimization, and evaluation. Each phase of the MOST is comprised of precise objectives that are tied to a series of supporting milestones, indented to aid in accomplishing goals that are structured to facilitate the development of an efficient, economic, and effective intervention design [85]. Several pilot studies have leveraged the MOST to examine the acceptability, feasibility, and preliminary effectiveness of behavioural interventions by employing a factorial design [87–89]. Using this method, researchers are able to identify components of an intervention that will produce the most optimal results and assess whether particular combinations would differ in terms of participation as certain components may contain materials that can create extra burden for participants [87]. Therefore, the MOST was applied in this study in service of (i) designing an efficient multicomponent online road safety education intervention for children, and (ii) evaluating the feasibility and preliminary effectiveness of the developed intervention.

**Table 1:** Outline of the MOST, adaptation to current study

Phase	Objectives	Associated milestone	Adaptation & educational objective
1 – Preparation	• Intervention conceptualization	• Developing conceptual models • Identification of potential candidate components (e.g., strategies, resources) • Conducting of pilot trials • Outlining of noteworthy constraints	• Conceptualize a multi-component OEI featuring material related to awareness, safe pedestrian travel behaviours, knowledge of signs, and cycling skill development. • *Objective(s)*: develop the structural outline of an OEI for parents and children to promote AT.
2 – Optimization	• Identifying the most efficient, effective etc. intervention structures or components that produce best outcomes	• Carrying out optimization trials (e.g., factorial trials) • Generating optimization criteria to assess the multicomponent intervention regarding effectiveness, economy, efficiency, scalability etc.	• Develop and refine module to be optimized for target intervention samples (i.e., parents and children), and generate analysis plan for the OEI evaluation. • *Objective(s)*: Collect and analyze relevant data and suggested materials for suitability and quality, and subsequently generate component modules for OEI.
3 – Evaluation	• Optimizing of intervention via evaluations of performance regarding stated outcome(s) (e.g., effectiveness, economy, efficiency, and/or scalability)	• Carrying out comprehensive trials • (Potential) dissemination of the optimized intervention	• Systematically test the effectiveness of the developed OEI with a multi-group design, and determine the most effective combination of modules to improve AT perceptions among parents and children. • *Objective(s)*: evaluate the effectiveness of the OEI regarding changing child and parental scores related to Active Travel Knowledge, Pedestrian Safety and Skills, Signs and Infrastructure knowledge, and Wheeling Safety and Skills.

Education-based AT interventions can be an important mechanism to support AT, however, more rigourous program designs and evaluations have been highlighted as being needed. The present study was undertaken to support the Active and Safe Routes to School (ASRTS) committee’s STP initiative by developing an intervention that sought to address the “education” and “evaluation” aspect of the AST Five E’s framework (i.e., Education, Encouragement, Engineering, Enforcement, Evaluation) [90].Therefore, while applying the MOST to guide the development and evaluation of a comprehensive multicomponent online road safety education intervention for children, the aim of this study was threefold:

1. To examine the feasibility of the *preparation* (i.e., design process, acceptability) and *optimization* (i.e., retention, scalability) of an online road safety education intervention among a sample of children and parents. (RQ1)
2. To *evaluate* the preliminary effectiveness of the online road safety education intervention on AT and road safety knowledge (i.e., pedestrian safety skills, wheeling skills, signs and infrastructure literacy) of children. (RQ2)
3. To *evaluate* the preliminary effectiveness of the online road safety education intervention on the AT practices and perceptions of parents. (RQ3)

## Methods

### Recruitment and sample

Participants for this study were recruited between January to June 2023 via a combination of digital (e.g., twitter ads) and traditional sampling (e.g., social networks) techniques. However, most participants were recruited from an ongoing online survey study being run by Western University that examined perceptions and behaviours related to school travel among a national sample of Canadian children and parents. This survey was facilitated by the Canadian market research firm Léger (Montreal, PQ, Canada) and was ultimately completed by roughly 1,500 participants. In the online survey, our team included a recruitment message that was displayed upon each participant’s completion. Our recruitment message prompted potentially interested participants to email the research team for more information regarding how to participate in this online road safety education intervention study. In tandem with this digital recruitment, our team also carried out more traditional recruitment methods which included snowball sampling via contacting potential participants within our team’s social and professional networks. As the intervention was developed for children and parents, families were deemed as being eligible for participation if they met the following criteria: (1) reside in Ontario, Canada^1^, (2) have a child between the ages of 9–13 years (i.e., child is old enough to have some level of independent mobility to engage in AT), (3) be part of a parent-child dyad (i.e., both parent and child are willing to enroll and complete intervention modules), and (4) have reliable internet access (intervention was delivered through an online platform). To be enrolled in the study, parents/guardians and children had to complete the family and youth baseline surveys, respectively, to provide written consent/assent and assess children’s baseline AT and road safety knowledge. Among the 134 parent-child dyads that were screened, 16 were ineligible and 35 did not complete the child baseline survey. Ultimately, a total of 83 dyads were enrolled in the study. Full sample demographics are presented in Table 2. Additional characteristics (e.g., school commuting behaviour, physical activity behaviour) for children who have completed the OEI are broken down into their respective groups (Additional File: Table S4).

**Table 2:** Full sample characteristics for the online road safety education intervention

Child characteristics, *n*	Initial sample (*n* = 83)	Final sample (57)
**Age in years**, mean (std)	10.1 (0.9)	10.1 (0.9)
**Grade**, mean (std)	4.9 (0.8)	4.8 (0.9)
**Gender**, n (%)		
Boy	45 (54.2)	35 (62.4)
Girl	36 (43.4)	20 (35.7)
Non-Binary	2 (2.4)	1 (1.8)
**Race**, n (%)		
Black	2 (2.4)	1 (1.8)
Caucasian	48 (57.8)	31 (55.4)
East & Southeast Asian	13 (15.6)	9 (19.7)
Latinx	2 (2.4)	1 (1.8)
South Asian	6 (7.2)	5 (8.9)
West Asian	1 (1.2)	1 (1.8)
Mixed Race	9 (10.8)	6 (10.7)
Prefer Not to Answer	1 (1.2)	0 (0)
**Immigration Status**, n (%)		
Born in Canada	79 (95.2)	53 (94.6)
Born Outside of Canada	3 (3.6)	3 (5.4)
Prefer Not to Answer	1 (1.2)	0 (0)
*Regions		
Central Ontario (non-GTA)	4	3
Central Ontario (GTA)	46	34
Eastern Ontario	4	3
Western Ontario	26	14
Northern Ontario	2	2
**Parent / Guardian Characteristics, n**	**Initial Sample (** ***n*** ** = 82)**	**Final Sample (56)**
**Gender**, n (%)		
Man	21 (25.6)	14 (25.5)
Woman	61 (74.4)	41 (74.5)
**Highest educational attainment**, n (%)		
High School	5 (6.1)	4 (7.3)
Apprenticeship / Trade Certificate	7 (8.5)	5 (9.1)
Post Secondary	52 (63.4)	34 (61.8)
Post Graduate Program	18 (22.0)	12 (21.8)
**Children live in two homes**, n (%)	5 (6.0)	4 (7.1)
**Children live in single-parent home**, n (%)	13 (15.7)	10 (17.9)

*Notes* Initial sample excludes participants who did not access the modules, whereas the final sample excludes participants who did not access the modules and did not complete the post-intervention survey. The child’s racial identity and immigration status were identified by the parent in the Family Baseline Survey. Additionally, one parent-child dyad contained two children who were enrolled in the study. | *Home postal codes were geocoded using the 2020 archive and further categorized into regions using the boundaries identified by the Ontario Public Service Region |

**Acronyms** GTA – Greater Toronto Area

### Study protocol

**Fig. 1:**
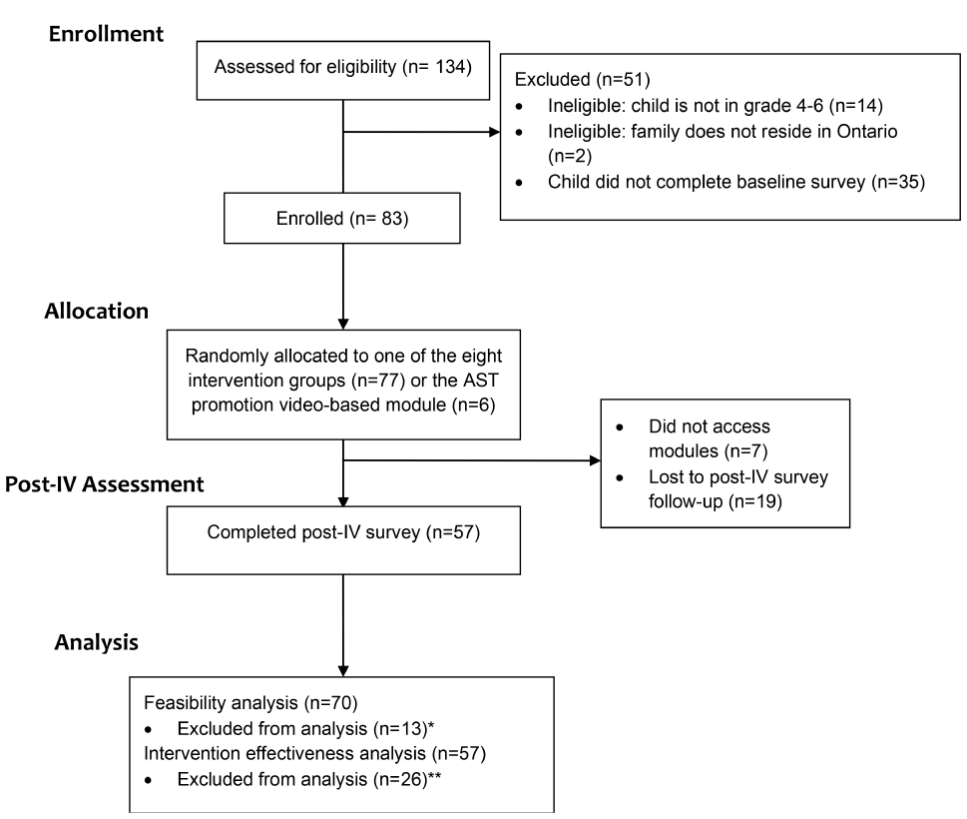
Study protocol flowchart. *Notes:* *Feasibility analyses excluded the control group (*n* = 6) and individuals who did not access the online modules (*n* = 7; i.e., not exposed to any condition). **Pre-post analyses excluded individuals who did not complete the post-IV survey. AST – active school travel; IV – intervention

Our protocol began with participants being randomly allocated, via a stratified (i.e., by gender, aim 50/50) sorting method, into one of the intervention experiment conditions or the control group (Fig. 1). Prior to engaging with their respective module(s), participants first completed a baseline survey on Qualtrics that collected their sociodemographic information, and then subsequently examined their awareness of active school travel (AST) benefits, road safety knowledge, and perceptions of AST barriers and facilitators. Survey questions were derived from our team’s validated Perceived AST Barriers and Enablers—Child (PASTEB-C) questionnaire [91]. Specific measures used in the survey to evaluate the intervention are detailed in the sections below. Next, participants were instructed to create an account on the Western University’s learning management system, OWL, to access their respective learning modules and materials. Any participants who had difficulties accessing their materials were aided by a research assistant (RA) on the research team. RAs also sent out reminder emails throughout duration of the study to ensure the continued participation of all individuals, and to mitigate potential conditions for dropout. Participants were asked to complete their assigned module(s) in 4-day intervals to ensure standardization. Therefore, participation ranged from 4 to 16 days depending on the condition(s) participants were assigned to (i.e., the number of modules they were asked to complete). Participants received a $30 gift card upon completion of the program.

Within each module, or experimental condition, participants were assigned a combination of readings, videos, quizzes, and activities (Additional File 1: Table S1). Upon completion of their respective module(s), participants were encouraged to provide feedback on each module (e.g., how engaging, enjoyable, informative etc. the content was) by filling out a feedback evaluation form. After completing the program, parents and children proceeded to complete a post-intervention survey that consisted of the same questions as the baseline survey, along with a set of additional open-ended questions probing participants about their overall satisfaction with the intervention and its delivery.^2^ Like the baseline survey, follow-up surveys were administered via Qualtrics. Ethics for the study and its protocols were approved by Western University’s non-medical research ethics board (application #121,096).

### Study design

In accordance with the MOST’s *optimization* phase guidelines, a single-blind (i.e., participants unaware of grouping and research question) 2^3^ (i.e., two-level [whether a module is present or not], three factors [number of modules being analyzed]) factorial controlled clinical design was used to investigate the impacts of each module component, resulting in eight distinct experimental conditions. To allow for a comprehensive analysis of the potential module-specific impacts, an additional control group was added. Participants in this condition received a standard video-based AST promotion module which consisted of testimonials covering what AST is and ways to engage, pro-AST messaging (e.g., fewer vehicles in school area, opportunities to socialize), and awareness of the health and environmental benefits of AST participation. Control participants did not receive any of the developed intervention’s modules.^3^ Conversely, the developed road safety intervention was comprised of a combination of four learning module components: Active Travel Knowledge (module 1), Pedestrian Safety and Skills (module 2), Signs & Infrastructure (module 3), and Wheeling Safety & Skills (module 4). This set of four modules was the result of a co-development process undertaken with numerous AST experts and practitioners who participated in a preceding study to this one [92]. With each intervention condition receiving the active travel knowledge module (i.e., module 1) to help standardize the analysis across these groups (i.e., the non-control groups), each condition had an additional set of specific modules to complete. The inclusion (‘ON’) or absence (‘OFF’) of modules for each experimental condition is outlined in Table 3.

**Table 3:** Eight experimental conditions implemented using a 2^3^ factorial design

Experimental condition	Active travel knowledge module	Pedestrian safety and skills module	Signs and infrastructure module	Wheeling safety and skills module
1	**[X]**	[—]	[—]	[—]
2	**[X]**	**[X]**	[—]	[—]
3	**[X]**	[—]	**[X]**	[—]
4	**[X]**	[—]	[—]	**[X]**
5	**[X]**	**[X]**	**[X]**	[—]
6	**[X]**	**[X]**	[—]	**[X]**
7	**[X]**	[—]	**[X]**	[**X**]
8	**[X]**	**[X]**	**[X]**	**[X]**

**Notes**: [X] – Used/ON | [—] – Absent/OFF

### Intervention structure and materials

To satisfactorily achieve the objective of the *preparation* phase of the MOST, the development of the intervention and its component modules was completed through collaborations with multiple key stakeholders [92]. The overarching goal of the intervention was to increase children’s knowledge and attitudes towards AT, which are prerequisites for behaviour change [93]. First, modules were organized into their respective constructs (i.e., Active Travel Knowledge, Pedestrian Safety and Skills, Signs and Infrastructure, and Wheeling Safety and Skills), which were developed and conceptualized by an integrated knowledge translational approach [92]. To incorporate different learning mediums (e.g., visual, auditory, reading and writing, and kinesthetic), each module contained readings, videos, activities, and quizzes (Additional File 1: Table S1). Tools and resources from various organizations across Ontario (e.g., AST committees, AST programming partners, and transportation services) were utilized in the content development of the modules. Readings, videos, and quizzes were compiled and reformatted into an eLearning course using Articulate Storyline 360, a cloud-based tool that allows instructors to design interactive online courses.

### Measures and analyses

#### Preparation: measures and analysis (RQ1)

Regarding the first aspect of RQ1, intervention preparation, this component of the program includes those milestones related to program design and acceptability [86], the latter specifically referring to how recipients of a program react to the proposed intervention [94]. In the context of this study, program design and acceptability were assessed through participant satisfaction via performing inductive content analysis (ICA) on the written responses provided in the module feedback evaluation forms and post-intervention surveys. Module feedback evaluation consisted of two open-ended questions regarding children’s favourite and/or least favourite part of the module(s) and how the modules can be improved for future implementation.^4^ Meanwhile, the post-intervention survey asked children and parents about what they liked and disliked regarding the overall program, and whether it has equipped them with the skills to increase or continue their use of active transportation. ICA primarily focuses on allowing themes and concepts to emerge from raw data (e.g., transcripts, written responses) without relying on previous knowledge and assumptions on the data [95]. Using Microsoft Excel, repeating responses were organized and coded into their respective themes. When themes emerged, researchers reviewed and recoded responses as needed. This iterative process allowed the research team to conceptualize the larger themes into subthemes, making it easier to identify the similarities and differences between groups.

#### Optimization: measures and analyses (RQ1)

With respect to the second aspect of the RQ1, intervention optimization, this phase of the MOST process involves those milestones related to retention and scalability [86]. These milestones were examined using basic statistical methods including computing percent retention and participation rates. Percent retention was measured by dividing the number of participants who were present at post-intervention (i.e., completed post-intervention survey) by the number of children who were present at enrollment. Participation rates were assessed by calculating the task completion rates (i.e., interactive module portion [readings, videos, and quizzes], online activities, and hands-on activities) for each component across the eight experimental groups. Percent retention and participation rates were calculated using Microsoft Excel.

#### Evaluation: preliminary effectiveness measures and analyses (RQ2, RQ3)

With respect to RQ2, this component of the intervention was evaluated using a variety of statistical methods. All measures used to evaluate the preliminary intervention effectiveness in children were drawn from a validated road safety knowledge questionnaire developed for AST programming [91]. The 26-item knowledge questionnaire was comprised of four AT knowledge questions and 22 road safety knowledge questions. Knowledge scores were collected at pre- and post-intervention to determine which group would experience a short-term knowledge change. The number of correct responses for each section were calculated, resulting in a final score range of 0–4 and 0–22 for AT knowledge and road safety knowledge, respectively.

Similarly, RQ3 was addressed via statistical analyses of items extracted from the validated Perceived Active School Travel Enablers and Barriers (PASTEB—P) parent questionnaire and the Physical Activity Parenting Practices (PAPP) item bank [96, 97]. The PASTEB—P questionnaire measured two types of parental AST perceptions (e.g., parental controls and perceived AT barriers). The PAPP item bank is a validated tool featuring multiple constructs regarding the PA practices of parents, specifically as they concern parental motivations, values, permissive (e.g., little to no parental guidance in child decision-making processes) and coercive influences (e.g., criticizing, nagging), and praise reward factors (e.g., reinforcement, reasoning) among other areas. Previously the PAPP has been used to examine related topics such as the moderating role of activity style on the relationship between parental practices and child moderate-to-vigorous physical activity [98], as well as in evaluations of physical activity literacy interventions [99]. In this study, the PAPP was used to assess parental AT practices via the following constructs: Guided choice, non-directive support, supportive expectation, and autonomy support [97].

Responses to all questionnaire items were collected at pre- and post-intervention to determine which experimental condition(s) would exhibit improvements regarding parental AT practices and perceptions. Parental control items were recorded as dichotomous responses (e.g., Yes, No), perceived barrier items were presented as 4-point Likert scale questions ranging from ‘strongly disagree’ to ‘strongly agree’, and the PAPP items were presented using a 5-point Likert scale, to which participants were asked to indicate how often they engaged in a particular behaviour or practice (1-Never, 2-Rarely, 3-Sometimes, 4-Often, 5-Very Often). A detailed outline of the parental AT practices and perceptions constructs is located in Additional File 1.

Due to the pilot nature of our study, intervention effectiveness could not be evaluated with full statistical power to detect differences. However, preliminary analyses were conducted to demonstrate the intervention’s potential to improve knowledge and perceptions, as it would be difficult to transition from a pilot study to a full-fledged powered study without observing the intervention’s influence on the outcome measures [88, 89]. Thus, findings should be interpreted as exploratory to inform future studies of larger scale. One-way ANOVAs were run to determine whether there were within-subject and between-subject variability by comparing age, gender, baseline AT knowledge, and baseline road safety knowledge test scores between groups. Paired-samples t-tests were used to assess the differences between the pre- and post-test knowledge scores for each group. A two-tailed independent samples t-test was performed to analyze the individual module effects on the post-test knowledge scores. The same tests were run for the parental outcome measures. Levene’s test for equality of variances supplemented the independent samples t-tests to determine the assumption of equal variances between the samples. All statistical analyses were completed on IBM SPSS (Version 25), with significance defined as *p* < 0.05.

## Findings

The findings of this study are presented according to the three research questions posed: the preparation, optimization (RQ1), and effectiveness (RQ2, RQ3) of the OEI. Despite the study not being powered to evaluate intervention effectiveness, findings regarding the program’s effectiveness are presented via a series of exploratory quantitative analyses to determine the preliminary effectiveness of the different modules and arranged groupings.

### Preparation (design process, acceptability)

From the ICA, responses were broken down into pros and cons, which refer to the positive and negative aspects of each module, respectively (Additional File 1: Table S2). As a result, 79 codes were generated for the cons category, which was further classified into six major themes: activities, videos, quizzes, module instructions, readings, and user interface, and nine subthemes. The former three, or the most prominent, themes are discussed below in reference to their positive and negative feedback.

#### Positives of implementation strategy

Of the themes generated related to the implementation of the intervention, ‘activities’ was predominant with 42 codes for the pros category. Of particular note among the activities was the AT Story (module 1: AT Knowledge) which was highlighted as an important engagement mechanism that participants seemed to enjoy due to its personalization quality. One child elaborated on this explaining that their “favourite part [of the module] was writing the [active travel] story” because it allowed them “to be super creative.” In line with this sentiment, a parent expressed “my favourite module was the active travel knowledge module because I really like the Hop activity and the active travel passport”. A second notable activity that participants found engaging was the bike rodeo (module 4: Wheeling Safety and Skills). Here, several participants expressed that they thoroughly enjoyed the Bike Rodeo due to its interactive nature, as one participant detailed that their “favourite part of the module was the bike rodeo…when I got out my bike again, it was very fun.”

Two additional major themes related to the implementation of the intervention included the general experience of the ‘videos’ presented throughout the program, and the impacts of the evaluation quizzes. Regarding the former, the videos were reputed to be informative as children frequently pointed out such sentiments as they “were fun to watch and provided good information.” More precisely, two children found the pedestrian crossover and crosswalk videos involving Lego figurines to be the most interesting and even suggested to add “more Lego videos” to improve the appeal of the intervention to this demographic. With respect to the quizzes, multiple participants genuinely found the quizzes enjoyable and informative, precisely due to their relatable questions, to which one participant suggested to “make more quiz questions”.

#### Issues with implementation strategy

Sentiments pertaining to the ‘activities’ theme were also critical. Consequential currents within this theme involved concerns related to activity difficulty, engagement, and duration, among other concerns. Common sentiments here saw participants express thoughts regarding some of the activities included in the different modules being too difficult to complete, notably the Mapping Your Route to School activity from module 1 (e.g., per one child, “the active map activity was difficult and frustrating”). In addition to the above concerns, other sentiments focused on the need for parental support for completion, with another child detailing, “I didn’t like the complicated mapping part…my mom helped so much I couldn’t have done it without her.” As a result, participants often pointed out that activities would take longer than expected (e.g., one parent commented, “we didn’ t have time to finish the activities because too much work”), to which one child suggested “making the activities shorter” would help with effective engagement. Moreover, some suggested that they would have enjoyed more gamified and/or hands-on activities to enhance enjoyment: “The game was my favourite part [of module 2] as it was fun…more games would make [the module] more fun and easy” and “my favourite part [of module 4] was the bike rodeo…maybe more hands on activities [can improve the module].” However, it should be noted, many individuals did not partake in the hands-on activities (e.g., Bike Rodeo, iSpy Signs, Road Safety Scavenger Hunt, etc.) due to scheduling conflicts and weather.

Like the ‘activities’ theme, the ‘videos’ and ‘quizzes’ were also met with mixed feelings. As it concerns the ‘videos’ theme, several children articulated that the number of videos presented throughout the program was a lot to digest, resulting in one participant stating their “least favourite part about [module 2] was that there were too many videos”. In line with this, another individual added that we should include “more interesting videos for [module 3]”, suggesting that video content should be more engaging to pique children’s interest. Also adding an engagement barrier was the legibility of some videos. Specific instances of this were mentioned in reference to module 3 (Signs and Infrastructure), where participants noted that it was difficult to depict what was said in one of the videos due to the audio’s volume, thus, future revisions should consider adding captions to increase accessibility. With respect to the ‘quizzes,’ the overall design of the quiz and difficulty of the questions were suggested to be inhibitive. Participants suggested that we add “more quiz questions” and implement an option where they can have several answer attempts so that “people [can] correct themselves if they get the wrong answer on the quiz.” From a pedagogical perspective, this approach would encourage children to learn from their mistakes, which can ultimately motivate them to be more inclined to retain the information from the quizzes.

### Optimization (retention, scalability)

#### Program retention and participation

Of the 83 parent-child dyads that initially enrolled, 57 completed the post-intervention survey, resulting in an overall retention of 69%. Removing the control group, which had complete retention, the different intervention groups reported considerably varying levels of retention ranging from 55 to 100%. Specifically, the participant retention breakdown for the individual groups were as follows: 100% (Control), 24% (Group 1), 67% (Group 2), 67% (Group 3), 64% (Group 4), 55% (Group 5), 67% (Group 6), 100% (Group 7), 78% (Group 8).

Table 4 presents the participation rates for each intervention module. Overall, completion rates across the four modules were fair, with 63-81% of the participants completing the interactive lessons (i.e., readings, videos, quizzes), 61-69% completing the online activities, and 40-51% completing the hands-on activities.

The factorial design allowed us to assess children’s participation across the various module combinations by analyzing the completion rates with respect to group assignment. While module 1 did not demonstrate any notable trends, interactive effects between modules 2, 3, and 4 were observed. When module 3 was presented in tandem with module 4 (i.e., group 7), overall completion rates for both modules were higher compared to when they were presented with module 1 alone (i.e., group 3 and 4). Interestingly, when module 2 was presented alongside module 3 (i.e., group 5), both modules exhibited lower overall completion rates compared to when they were presented as standalone components with module 1 (i.e., groups 2 and 3). It is worth mentioning that the grouping of modules 2, 3, and 4 in group 8 illustrated positive interactions across the three modules, particularly, the online activities.

**Table 4:** Participation rates for each module across all intervention (IV) groups

IV Group	*N*	Active travel knowledge (*n* = 70)			Pedestrian safety and skills (*n* = 35)			Signs and infrastructure (*n* = 32)			Wheeling safety and skills (*n* = 35)		
		**Interactive Lesson**	Online Activities*	Hands-on Activities*	**Interactive Lesson**	Online Activities*	**Hands-on Activities**	**Interactive Lesson**	**Online Activities**	**Hands-on Activities**	**Interactive Lesson**	Online Activities*	**Hands-on Activities**
1	11	6/11 (54%)	13/22 (59%)	8/22 (36%)	X	X	X	X	X	X	X	X	X
2	8	6/8 (75%)	9/16 (56%)	7/16 (44%)	5/8 (63%)	8/16 (50%)	5/8 (63%)	X	X	X	X	X	X
3	7	6/7 (86%)	11/14 (79%)	8/14 (57%)	X	X	X	6/7 (86%)	5/7 (71%)	5/7 (71%)	X	X	X
4	10	7/10 (70%)	14/20 (70%)	13/20 (65%)	X	X	X	X	X	X	7/10 (70%)	10/20 (50%)	2/10 (20%)
5	9	6/9 (67%)	14/18 (78%)	8/18 (44%)	6/9 (67%)	11/18 (61%)	4/9 (44%)	6/9 (67%)	5/9 (56%)	3/9 (33%)	X	X	X
6	9	6/9 (67%)	13/18 (72%)	13/18 (72%)	4/9 (44%)	13/18 (72%)	5/9 (56%)	X	X	X	6/9 (67%)	12/18 (67%)	3/9 (33%)
7	7	7/7 (100%)	8/14 (57%)	5/14 (36%)	X	X	X	7/7 (100%)	5/7 (71%)	5/7 (71%)	7/7 (100%)	10/14 (71%)	4/7 (57%)
8	9	7/9 (78%)	15/18 (83%)	9/18 (50%)	7/9 (78%)	11/18 (61%)	4/9 (44%)	7/9 (78%)	7/9 (78%)	6/9 (67%)	7/9 (78%)	11/18 (61%)	5/9 (56%)
Total	70	51/70 (73%)	97/140 (69%)	71/140 (51%)	22/35 (63%)	43/70 (61%)	18/35 (51%)	26/32 (81%)	22/32 (69%)	19/32 (59%)	27/35 (77%)	43/70 (61%)	14/35 (40%)

**Notes**: The interactive lesson includes the reading, video, and quiz components. | Components with two activities are marked with an asterisk (*)

#### Scalability

Regarding the scalability (i.e., expansion in usage to other contexts), there were two central themes that emerged from the qualitative analysis of participant feedback on the overall program: improved knowledge, and behaviour change (Additional File 1: Table S3).

Responses pertaining to children’s improved understanding of multiple AT topics were provided to materials used across the program. Children across the eight groups, and particularly groups 2 and 4, expressed that they benefited from engaging with various materials of the intervention, namely due to the focus on *sign and infrastructure literacy and* ‘*rules of the road*’ topics, with participant explanations noting, “I now know what the signs are for and what the rules are to safely use the road as a pedestrian” and “[the program] taught me more safety skills for my bike.” Similarly, a parent from group 6 commented on their child’s increased knowledge as they now “understand the value of biking”. Not only did participants learn more about the various pedestrian responsibilities and rules on the road, but several added that they now better understand the *importance of AT and road safety*. Specific examples of this included feedback highlighting new perspectives of how signs give specific information to drivers/pedestrians/cyclists about the road, and AT being a relatively easy way to be physically active. Taken together, the intervention’s materials were suggested to improve child knowledge through enhancing their conceptualizations of road safety, improving child understandings of the ‘rules of the road’ (e.g., pedestrian responsibilities, signage identification and associated behaviours), and increasing participant knowledge of AT opportunities/accessibility. Feedback regarding the various foci of the four different component modules, overall, suggested that the OEI has good potential regarding its scalability.

In addition to the potential to replicate increased road safety and AT knowledge, a secondary scalability theme was the intervention’s impact on perceptions of behaviour changes, and specifically materials targeting intrinsic motivation pertaining to actively commuting to school. Representative of this feedback, various children explained, post-intervention, that “[the program] tells me the benefits which makes me want to walk to school more,” and “[the OEI] made me want to bike to school,” and “[the OEI] helped me be prouder of biking.” In a similar sentiment, one parent stated, “it has helped me see I should let him use his bike”. Another parent noted, “[the program] was definitely helpful in making [my child] more aware of her impact on the environment”, further suggesting that our program has positive implications on environmental awareness. Across the feedback from participants in different intervention groups, there was a similar sentiment regarding these motivations regarding AT reportedly extending beyond the child participants. For example, one child observed “[the OEI] made my dad change his mind [about increasing the use of active transportation] a little bit,” which coincided with another parent’s comment “I would say it has equipped us with the skills to continue to use active transportation. I will try to get them to walk even more”. These sentiments illustrate the potential of the intervention to drive larger family motivation for changing AT behaviours. In sum, feedback regarding the materials of the OEI were also indicated to have considerable potential to promote AT behaviour change through fostering motivation related to supporting the development of responsible pedestrian road skills (e.g., pedestrian crossing techniques, bicycle maneuvers) and pro-AT attitudes (e.g., benefits of AT, parental encouragement/permission).

### Evaluation (changes in perceptions, knowledge)

#### Preliminary intervention effectiveness for children

There were no statistical differences between the groups with respect to age (F [8, 48] = 0.899, *p* = 0.525), grade (F [8, 47] = 1.094, *p* = 0.384), road safety knowledge scores (F [8, 48] = 0.068, *p* = 1.000), and AT knowledge scores (F [8, 48] = 0.435, *p* = 0.894) at baseline.

Paired-samples *t* test and two-tailed independent-samples *t* test results pertaining to road safety knowledge and AT knowledge scores are presented in Tables 5 and 6, respectively. All intervention groups, as well as the control group, demonstrated some level of improved AT knowledge from baseline to post-intervention evaluation, with group 6 seeing the largest gains (mean difference: 1.00 [1.45], *p* = 0.152). However, none of these improvement scores were statistically significant. With respect to road safety knowledge scores, all groups with the exception of group 1 (mean difference: -0.46 [2.51], *p* = 0.674) showed a little-to-moderate improvement in their scores from pre-to-post-intervention. Here, the changes in scores for group 4 (mean difference: 3.96 [2.17], *p* = 0.003), group 6 (mean difference: 3.30 [2.77], *p* = 0.033), group 7 (mean difference: 3.79 [2.16], *p* = 0.004), and group 8 (mean difference: 3.94 [2.85], *p* = 0.011) were statistically significant, which corresponds to the significantly better mean post-intervention road safety knowledge scores for groups that completed the wheeling safety and skills module than those who did not complete this module (mean difference: 2.39, *p* = 0.004).

**Table 5:** Mean road safety knowledge and AT knowledge scores from pre-intervention to post-intervention

Condition	*N*	Road Safety Knowledge				Active Travel Knowledge			
		Mean Pre-IV Score (SD)	Mean Post-IV Score (SD)	Difference of mean scores	p value^a^	Pre-IV Score (SD)	Post-IV Score (SD)	Difference of mean scores	p value^a^
Control	6	13.59 (2.56)	14.76 (2.53)	1.17 (1.45)	0.106	2.42 (0.80)	3.00 (0.63)	0.58 (0.80)	0.135
IV Group 1	6	14.27 (2.87)	13.81 (4.88)	-0.46 (2.51)	0.674	2.67 (0.98)	2.83 (0.75)	0.17 (0.52)	0.465
IV Group 2	6	14.67 (1.89)	16.61 (2.13)	1.94 (2.14)	0.077	2.50 (0.84)	2.92 (0.49)	0.42 (0.49)	0.093
IV Group 3	6	13.99 (3.56)	15.49 (2.90)	1.50 (3.42)	0.332	2.33 (1.21)	3.00 (0.00)	0.67 (1.21)	0.235
IV Group 4	7	14.36 (4.33)	18.32 (3.21)	3.96 (2.17)	**0.003**	2.71 (0.49)	3.00 (0.00)	0.29 (0.49)	0.172
IV Group 5	6	14.33 (2.06)	16.66 (2.34)	2.33 (3.10)	0.125	2.67 (1.03)	3.17 (0.41)	0.50 (1.22)	0.363
IV Group 6	6	14.50 (4.42)	17.80 (2.37)	3.30 (2.77)	**0.033**	2.00 (1.45)	3.00 (0.00)	1.00 (1.45)	0.152
IV Group 7	7	14.22 (2.14)	18.01 (2.21)	3.79 (2.16)	**0.004**	2.64 (0.85)	3.07 (0.19)	0.43 (0.79)	0.200
IV Group 8	7	14.00 (2.01)	17.94 (1.98)	3.94 (2.85)	**0.011**	2.14 (0.90)	2.43 (1.13)	0.29 (1.70)	0.673

**Notes**: Control – AST promotion video-based module; **Group 1** – AT module ONLY; **Group 2** – AT module + Pedestrian Road Safety module; **Group 3** – AT module + Signs and Infrastructure module; **Group 4** – AT module + Wheeling Safety and Skills module; **Group 5** – AT module + Pedestrian Road Safety module + Signs and Infrastructure module; **Group 6** – AT module + Pedestrian Road Safety module + Wheeling Safety and Skills module; **Group 7** – AT module + Signs and Infrastructure module + Wheeling Safety and Skills module; **Group 8** – AT module + Pedestrian Road Safety module + Signs and Infrastructure + Wheeling Safety and Skills module. Acronyms: IV – Intervention. | **Bold** = significant at 0.05 level. | Results should be interpreted as exploratory due to insufficient power

**Table 6:** Mean road safety knowledge and AT knowledge post-test scores for each ‘ON’ and ‘OFF’ component

	Intervention Components			
		**Pedestrian Safety and Skills Module**	**Signs and Infrastructure**	**Wheeling Safety and Skills**
Post IV Road Safety Knowledge Scores, Mean (SD)	OFF	16.54 (3.70)	16.70 (3.58)	15.64 (3.25)
	ON	17.28 (2.15)	17.10 (2.45)	18.03 (2.35)
	Difference of mean scores	0.73	0.40	2.39
	p-value	0.388	0.645	**0.004**
Post IV Active Travel Knowledge Scores, Mean (SD)	OFF	2.98 (0.36)	2.94 (0.42)	2.98 (0.48)
	ON	2.86 (0.70)	2.90 (0.66)	2.87 (0.61)
	Difference of mean scores	0.12	-0.04	-0.11
	p-value	0.440	0.818	0.487

**Notes**: Active Travel Knowledge module component was not examined due to being present in all the intervention conditions. Pedestrian Safety and Skills Module: ON (*n* = 25), OFF (*n* = 26); Signs and Infrastructure Module: ON (*n* = 26), OFF (*n* = 25); Wheeling Safety and Skills Module: ON (*n* = 27), OFF (*n* = 24). Difference of mean scores = mean (ON) – mean (OFF). Equal variance was assumed for all calculations, except for the Pedestrian Safety and Skills module as determined by Levene’s Test. | **Bold** = significant at 0.05 level. | Results should be interpreted as exploratory due to insufficient power

#### Preliminary intervention effectiveness for parents

One way ANOVA showed no statistical differences between groups with respect to the baseline PAPP measures: non-directive support (F [8, 48] = 0.795, *p* = 0.609). supportive expectations (F [8, 48] = 1.119, *p* = 0.368), guided choice (F [8, 48] = 1.719, *p* = 0.118), and autonomy support (F [8, 47] = 1.633, *p* = 0.141). With respect to parental perceptions, statistical differences were observed at baseline for barriers to road safety (F [8, 36] = 2.314, *p* = 0.041), but not for parental controls (F [8, 48] = 1.920, *p* = 0.079).

Paired-samples *t* test and two-tailed independent-samples *t* test results pertaining to parental PAPP, AT perceptions and practices are presented in Tables 7 and 8, respectively. No notable patterns were observed across the intervention groups regarding parents’ non-directive support, however, the control group demonstrated a significant decrease from baseline (mean difference: -1.67 [1.03]). All groups, including the control group, demonstrated little to no change in parental supportive expectation and guided choice, except for group 8, which exhibited a slight, but significant, improvement in the guided choice measure (mean difference: 0.86 [0.90], *p* = 0.045). Significant improvements in parental support for child’s PA autonomy were observed in groups 1 (mean difference: 1.83 [1.72], *p* = 0.048) and 6 (mean difference: 2.67[2.33], *p* = 0.038), but significantly declined for group 7 (mean difference: -2.71 [2.81]). With the exception of groups 1, 3, and 6, the extent to which parents can afford their child independent mobility opportunities (i.e., parental controls) have moderately increased, though insignificant. Interestingly, parents across all groups, except for groups 5 and 6, experienced a low to moderate reduction in perceived road safety barriers after completing the program, but significance was only observed in group 4 (mean difference: -2.67[0.58], *p* = 0.015). As illustrated in Table 8, parents who had children that were assigned to receive the wheeling safety and skills module had significantly better post-guided choice scores than those who did not receive this module (mean difference: 0.84, *p* = 0.032).

**Table 7:** Mean self-reported parental AT practices, and perceived controls and barriers scores from pre-intervention to post-intervention

Condition	*N*	Parental Self-Reported Outcomes (Score Range)							
		Non-directive Support (0–15)				Supportive Expectation (0–10)			
		Baseline Mean (SD)	Post-IV Mean (SD)	Difference of mean scores	p value^a^	Baseline Mean (SD)	Post-IV Mean (SD)	Difference of mean scores	p value^a^
Control	6	13.33 (1.50)	11.67 (2.16)	-1.67 (1.03)	**0.011**	8.33 (1.63)	8.33 (1.86)	0.00 (1.09)	1.000
IV Group 1	6	11.17 (2.79)	11.33 (2.66)	0.16 (2.56)	0.880	8.67 (1.63)	9.00 (1.26)	0.33 (0.52)	0.175
IV Group 2	6	10.50 (3.39)	9.00 (1.90)	-1.50 (1.97)	0.122	8.50 (1.64)	8.33 (1.37)	-0.17 (1.17)	0.741
IV Group 3	6	10.67 (2.42)	11.83 (3.13)	1.17 (2.32)	0.272	7.50 (1.52)	7.50 (1.97)	0.00 (2.19)	1.000
IV Group 4	7	11.57 (1.90)	10.00 (2.58)	-1.57 (2.07)	0.091	9.83 (0.41)	9.00 (1.26)	-0.83 (1.33)	0.185
IV Group 5	6	11.17 (2.56)	11.17 (2.13)	0.00 (2.53)	1.000	8.67 (1.21)	8.33 (1.37)	-0.33 (0.52)	0.175
IV Group 6	6	12.00 (2.28)	12.83 (1.94)	0.83 (1.72)	0.289	8.67 (1.63)	8.67 (1.63)	0.00 (2.19)	1.000
IV Group 7	7	12.29 (2.43)	11.00 (2.23)	-1.29 (1.97)	0.136	8.86 (1.57)	8.43 (1.81)	-0.43 (1.62)	0.510
IV Group 8	7	11.43 (2.15)	12.43 (2.70)	1.00 (1.63)	0.156	9.29 (0.76)	9.86 (0.38)	0.57 (0.79)	0.103

**Condition**	**N**	**Guided Choice (0–10)**				**Autonomy Support (0–20)**			
		Baseline Mean (SD)	Post-IV Mean (SD)	Difference of mean scores	p value^a^	Baseline Mean (SD)	Post-IV Mean (SD)	Difference of mean scores	p value^a^
Control	6	8.33 (1.63)	9.17 (1.17)	0.83 (1.33)	0.185	15.67 (3.67)	16.67 (4.18)	1.00 (2.10)	0.296
IV Group 1	6	7.68 (1.37)	8.00 (1.41)	0.33 (1.51)	0.611	17.00 (1.55)	18.83 (1.60)	1.83 (1.72)	**0.048**
IV Group 2	6	6.83 (1.72)	7.16 (0.98)	0.33 (1.86)	0.679	15.50 (2.88)	16.50 (3.08)	1.00 (2.44)	0.363
IV Group 3	6	7.17 (1.83)	7.67 (1.97)	0.50 (1.22)	0.363	15.33 (3.39)	15.17 (3.92)	-0.17 (3.43)	0.910
IV Group 4	7	8.86 (1.21)	8.29 (1.50)	-0.57 (1.27)	0.280	17.33 (2.66)	16.50 (2.59)	-0.83 (1.33)	0.185
IV Group 5	6	8.50 (1.52)	8.17 (0.98)	-0.33 (1.51)	0.611	17.00 (3.32)	15.80 (4.32)	-1.20 (3.03)	0.426
IV Group 6	6	8.17 (1.33)	8.50 (1.05)	0.33 (1.86)	0.679	16.00 (2.76)	18.67 (2.07)	2.67 (2.33)	**0.038**
IV Group 7	7	8.86 (1.57)	7.86 (1.81)	-1.00 (1.15)	0.062	18.57 (1.62)	15.86 (3.01)	-2.71 (2.81)	**0.043**
IV Group 8	7	8.86 (0.90)	9.71 (0.76)	0.86 (0.90)	**0.045**	19.00 (1.00)	19.00 (1.15)	0.00 (1.00)	1.000

**Condition**	**N**	**Parental Controls (0–13)**				**Barriers – Road Safety (0–16)**			
		Baseline Mean (SD)	Post-IV Mean (SD)	Difference of mean scores	p value^a^	Baseline Mean (SD)	Post-IV Mean (SD)	Difference of mean scores	p value^a^
Control	6	3.17 (2.14)	4.17 (4.26)	1.00 (4.81)	0.633	14.67 (2.52)	10.00 (3.60)	-4.67 (2.31)	0.073
IV Group 1	6	5.67 (2.94)	5.67 (3.56)	0.00 (0.89)	1.000	16.20 (5.07)	15.60(5.68)	-0.60 (2.51)	0.621
IV Group 2	6	2.67 (3.56)	5.83 (7.14)	3.17 (6.88)	0.311	17.50 (3.53)	14.00 (4.24)	-3.50 (0.71)	0.090
IV Group 3	6	4.83 (4.40)	4.50 (3.51)	-0.33 (4.84)	0.873	17.00 (1.79)	18.17 (2.23)	-1.17 (2.63)	0.328
IV Group 4	7	3.57 (2.23)	3.86 (3.08)	0.29 (2.63)	0.783	15.67 (1.15)	13.00 (1.00)	-2.67 (0.58)	**0.015**
IV Group 5	6	6.17 (3.13)	11.33 (4.13)	5.17 (6.17)	0.096	15.00 (4.05)	15.00 (2.76)	0.00 (2.00)	1.000
IV Group 6	6	6.83 (3.49)	6.00 (3.95)	-0.83 (2.23)	0.402	10.25 (4.65)	13.25 (2.99)	3.00 (3.55)	0.190
IV Group 7	7	6.29 (5.15)	11.00(8.21)	4.71 (7.61)	0.152	15.83 (2.48)	15.33 (2.42)	-0.50 (2.81)	0.681
IV Group 8	7	1.71 (1.38)	6.29 (5.91)	4.57 (5.79)	0.082	16.86 (4.01)	16.57 (3.82)	-0.29 (1.80)	0.689

**Notes**: Control – AST promotion video-based module; **Group 1** – AT module ONLY; **Group 2** – AT module + Pedestrian Road Safety module; **Group 3 -** AT module + Signs and Infrastructure module; **Group 4** – AT module + Wheeling Safety and Skills module; **Group 5** – AT module + Pedestrian Road Safety module + Signs and Infrastructure module; **Group 6** – AT module + Pedestrian Road Safety module + Wheeling Safety and Skills module; **Group 7** - AT module + Signs and Infrastructure module + Wheeling Safety and Skills module; **Group 8** - AT module + Pedestrian Road Safety module + Signs and Infrastructure + Wheeling Safety and Skills module. Acronyms: IV – Intervention. |**Bold** = significant at 0.05 level. | Results should be interpreted as exploratory due to insufficient power

**Table 8:** Mean parental AT practices, and perceived controls and barriers post-intervention scores for each ‘ON’ and ‘OFF’ component

	Intervention Components			
		**Pedestrian Safety and Skills Module**	**Signs and Infrastructure**	**Wheeling Safety and Skills**
Post Non-Directive Support Scores, Mean (SD)	OFF	11.00 (2.58)	10.76 (2.60)	10.83 (2.58)
	ON	11.40 (2.57)	11.61 (2.48)	11.52(2.53)
	Difference of mean scores	0.40	0.86	0.69
	p-value	0.581	0.235	0.344
Post Supportive Expectation Score, Mean (SD)	OFF	8.48 (1.63)	8.75 (1.33)	8.29 (1.52)
	ON	8.84 (1.34)	8.58 (1.65)	9.00 (1.41)
	Difference of mean scores	0.36	-0.17	0.71
	p-value	0.399	0.687	0.094
Post Guided Choice Score, Mean (SD)	OFF	7.96 (1.51)	8.00 (1.29)	7.75 (1.36)
	ON	8.44 (1.29)	8.38 (1.52)	8.60 (1.37)
	Difference of mean scores	0.48	0.38	0.84
	p-value	0.231	0.337	**0.032**
Post Autonomy Support Score, Mean (SD)	OFF	16.56 (3.07)	17.63 (2.52)	16.71 (3.38)
	ON	17.68 (2.86)	16.65 (3.36)	17.50 (2.59)
	Difference of mean scores	1.12	-0.97	0.79
	p-value	0.189	0.256	0.356
Post Parental Controls Score, Mean (SD)	OFF	6.35 (5.67)	5.28 (4.44)	6.83 (5.25)
	ON	7.32 (5.61)	8.30 (6.27)	6.81 (6.00)
	Difference of mean scores	0.97	3.03	-0.02
	p-value	0.541	0.052	0.991
Post Barriers to Road Safety Score, Mean (SD)	OFF	9.62 (3.32)	9.17 (3.67)	9.65 (3.42)
	ON	9.45 (3.13)	9.88 (2.73)	9.44 (3.06)
	Difference of mean scores	-0.16	0.72	-0.21
	p-value	0.865	0.434	0.822

**Notes**: Pedestrian Safety and Skills Module: ON (*n* = 25), OFF (*n* = 26); Signs and Infrastructure Module: ON (*n* = 26), OFF (*n* = 25); Wheeling Safety and Skills Module: ON (*n* = 27), OFF (*n* = 24). Difference of mean scores = mean (ON) – mean (OFF). Equal variance was assumed for all calculations, except for the Signs and Infrastructure measure for Parental Controls, as determined by Levene’s Test. **Bold** = significant at 0.05 level. | Results should be interpreted as exploratory due to insufficient power

## Discussion

In the present study, we recruited a sample of 83 parent-child dyads to evaluate the *feasibility*, via the preparation (i.e., design process, acceptability) and optimization (i.e., retention, scalability) of an OEI for road safety among children and parents (RQ1); the *preliminary effectiveness* of an OEI, via its impacts on children’s AT and road safety knowledge (i.e., pedestrian safety skills, wheeling skills, sign and infrastructure literacy) of children (RQ2), and parents’ AT practices and perceptions (RQ3). We applied the MOST to guide the development of our intervention approach. Central findings of this study include (i) both positive and critical sentiments regarding the design and implementation processes of the intervention, with most feedback centered on the program’s activities, videos, and quizzes (*preparation*); (ii) moderate participant engagement as reflected by program retention, but reputedly promising scalability based on feedback concerning the material used to improve AT knowledge and promote AT behaviour change (*optimization*); (iii) the OEI not significantly improving AT knowledge scores of children but, in some instances, significantly improving the road safety knowledge scores of children (*effectiveness*); and (iv) several occurrences of the OEI significantly improving some measures of parental AT practices and perceptions. Research, programming, and practical implications are discussed in more detail below, as are future research opportunities based on these findings.

Our feasibility analysis outlined mixed feelings among participants regarding the preparation and optimization of the intervention. For instance, feasibility concerns were observed through the fact that among the eight intervention groups, group 1 (module 1 only) experienced the highest drop-out rate and relatively low completion rates. From our ICA, it was suggested that these design process and acceptability outcomes related to the intervention were hampered by the selection of certain module activities. Primary concerns stemmed from one particular activity, *Mapping Your Route to School*, as several children expressed that it was difficult to complete the activity without parental support. A few parents have noted that activities in module 1 were time consuming, alluding to the difficulty of completing the module. The consistency of these concerns in our feedback would suggest that intervention activities could consider employing strategies with a greater emphasis on learning independence, especially as children who exhibit independence are more likely to have greater motivation, making them more engaged and receptive to the content [100]. Promoting child learning independence in the context of road safety education has the additional benefit of potentially contributing to positively influencing parents’ perceptions [101], a potential phenomenon that was alluded to in our qualitative analyses. Noting this, future research related to education-based AT interventions should consider the appropriateness of each modules’ activities with a particular focus on the independent learning potential of their employed strategies.

On the other hand, group 7 (modules 1, 3, and 4) exhibited the lowest drop-out rate and highest collective completion rates across the three modules, despite presenting more content and covering more topics than group 1. When considering potential explanations for these trends in our findings, it should be recounted that feedback in our qualitative analyses regarding scalability highlighted the combination of multiplicity of materials used and their varied aims. Such combinations which build connections between modules and content have been found to support children in successful active learning, a learning method that emphasizes cognitive engagement, ultimately resulting in increased student engagement [102, 103]. As it concerns the present study, the higher engagement observed in group 7 could be due to a combination of content relevance and comprehensiveness. Indeed, the three modules that group 7 interacted with could have thus satisfied the desire for information regarding prioritized areas related to AST (e.g., cycling knowledge), or relevance, and multiple activities to sustain engagement (e.g., drag and drop, matching traffic signs), or comprehensiveness. Put simply, the outline of group 7’s involvement in the intervention may reflect the most ideal structure to sustain engagement through the relevance (i.e., presenting priority information) and comprehensiveness (i.e., not insufficiently short nor ponderously long) of material presented. However, with the present paper detailing a pilot study evaluation, we encourage future work to continue exploring the most effectual module structure(s) for promoting AT and road safety knowledge in service of designing more engaging and optimized future interventions.

With respect to the evaluation of the preliminary effectiveness of the intervention and its various module combinations on children’s road safety knowledge, our findings indicated that groups that featured ‘wheeling safety and skills’ content (i.e., groups 4, 6–8) which included activities featuring bike equipment identification, bike checks, and bike safety and handling skill activities, reported significant knowledge improvements. One potential interpretation of this link is that a targeted focus on cycling skills is especially prioritized by children, resulting in higher levels of engagement and motivation. This was illustrated by one child’s feedback to which they pointed out that their favourite part of module 4 was the bike rodeo activity as it encouraged them to practice their cycling skills. Such a phenomenon could be the result of this content directly addressing cycling for transportation and related perceptions of knowledge and self-efficacy among children being consistently raised as a central barrier to AT [15]. Alternatively, the preliminary effectiveness of our intervention regarding road safety knowledge might derive from the multicomponent approach and/or specific strategies applied. Multicomponent intervention approaches, in related areas of study, have previously been reported to have some effectiveness with respect to PA promotion and education among children and adolescents [104], as well as PA education to specific groups like young girls [105]. More precisely, it could be that previously reported effective PA support strategies featuring multiple engagement activities demonstrating desired outcomes [106], or multiple complementary education sessions (e.g., addressing perceived barriers and engaging counter conditioning activities) tailored for a target group [107], were critical in this respect. While the current study only examined road safety and AT knowledge retention, future powered studies should consider implementing virtual reality or streetside methodologies to assess whether increased knowledge will translate into improved traffic safety and AT behaviours [19, 78, 108].

Conversely, evaluations of the effectiveness of the AT knowledge module and their inclusion among different groups and conditions did not result in any significant changes in scores among children. While scores were improved from baseline to follow-up, none of these changes were significant, a trend that may be due to a few different factors. Perhaps most effectual is the likely existing level of selection bias in our sample (i.e., participants expressed interest in this intervention based on participation in a separate AST survey) that predisposed our study towards the null hypothesis [109]. In the case of our intervention, it may thus be the case that a considerable number of participants already possessed high levels of knowledge pertaining to the general awareness of AST and its associated benefits (i.e., those topics which these modules sought to cover)—information which is often much less frequently reported as a barrier to AST than road safety knowledge [15]. Alternatively, it might be that while we applied the MOST to generate a relevant and scalable AST education intervention, we cannot rule out that the content of our module perhaps did not address the foremost areas of deficient AT knowledge among our specific sample. For instance, one area of general concern among children as it relates to general AT knowledge that is often reported is environmental or ecological impacts and benefits [110–112], which was more tangentially covered in our module 1 (AT Knowledge) as opposed to being a central theme throughout our program. Or third, our findings might be reflective of our activity selection. To supplement the educational content of this module (module 1), we opted to employ relatively simple user-driven activities such as geo-tracking web-apps and AT passports to monitor daily steps and commutes, activities which feedback suggested were received with mixed reviews. Perhaps more interactive methods like buddy programs [113], gender-specific strategies, or lifestyle-oriented approaches [114] which have been associated with improvements regarding PA education and behaviour change may have resulted in significant AT knowledge changes. Observing these trends across all of the evaluated cohorts and conditions in our intervention evaluation, we encourage future research to develop more targeted AT knowledge activities, especially as it concerns environmental considerations, gender-specific issues, and/or buddy programs in future intervention initiatives.

Evaluations pertaining to the preliminary effectiveness of the intervention on parental AT practices and perceptions have illustrated that no notable patterns or trends were observed for the PAPP measures such as non-directive support, supportive expectation, and guided choice across all intervention groups, with the exception of group 8, which exhibited a small, but significant improvement in the guided choice measure (i.e., employing approaches to support their child’s independence such as allowing their child to choose activities to do as a family). Interestingly, parents assigned to groups that featured the ‘wheeling safety and skills’ module have shown to report higher guided choice scores upon program completion compared to parents who did not receive this component. These results suggest that like children, parents valued and prioritized cycling skills education as it could help enhance risk awareness and promote child independence through increased safety conversations [78]. Our qualitative analyses on parental feedback displayed a similar sentiment with one parent adding that the program has made them more inclined to let their child bike to school. Studies that have examined parental perceptions on their child’s cycling skills have demonstrated that parental confidence is an important factor that influences cycling rates, to which it serves as a mediator between overall safety and cycling behaviour [42, 64]. Contrary to what was expected, group 7 exhibited a significant decline in parental support for child autonomy, while groups 1 and 6e significantly improved, suggesting that the ‘signs and infrastructure’ module may have negatively interacted with the ‘wheeling safety and skills’ module. This goes against the trend observed for our child road safety knowledge analysis, to which group 7 demonstrated a significant improvement in knowledge. An increase in child traffic safety knowledge has been shown to increase parental support for child autonomy, or more specifically, independent mobility, as their child would be more well equipped to behave safely on roads, thus reducing parents’ fear of traffic and social safety situations [15, 108]. Signage and infrastructure literacy is a critical component for cycling education as it supports the development of hazard perception skills (i.e., ability to read the road) through building an understanding of the various traffic structures and its associated behaviours, thereby, increasing the ability to detect dangerous situations on the road [17]. Future research should explore the reasons behind why an increased knowledge in road infrastructures and signage identification would result in a decline in parental child autonomy support, despite its importance on parental perceptions on children’s AT behaviour [115].

### Programming and practice implications

Results of this study have important insights for public health promotion, school health, and urban planning practitioners. As it concerns health promotion and school health activities, reviews of AST interventions have highlighted child education as an important programming consideration, and one which should be addressed when devising strategies to effectively improve child participation in AST [116]. Our developed OEI, or specific modules within it, could be used as an additional feature of such interventions (e.g., STP programming) to bolster the comprehensiveness and potential effectiveness of larger AST promotion initiatives targeting child road safety knowledge. For example, AST interventions must often consider in their programming ways to grapple with issues related to children’s signage and infrastructure literacy [117, 118], for which modules such as 2 and 3 from our OEI could be used to support school community aims pertaining to collision reduction and safe pedestrian and/or safe cycling behaviours among children. Beyond its use in different AT initiatives, our OEI, or some of its specific modules, could be used by planners to support higher-risk communities that lack quality pedestrian infrastructure related to public spaces that are frequented by children (e.g., school areas). Previous studies have highlighted that communities which lack adequate pedestrian infrastructure (e.g., bike lanes, pedestrian crossings) tend to see higher crash and injury risk compared to those with these designs [119], a trend which also appears to be true of built environments (e.g., lack of safe road crossings) around school areas [120]. Our OEI could be used in such vulnerable communities with comparatively worse pedestrian infrastructure to support improved pedestrian behaviour among children in service of reducing their risk of injury or motor vehicle-pedestrian collisions.

### Limitations

Findings from this study should be interpreted in light of the ensuing limitations. First, our module development was centrally based on collaborations with AST experts, law enforcement officials, school administrators, and transportation engineers with little input from children themselves. While we aimed to address identified ‘priority areas’ related to AT (e.g., cycling skills, sign identification), it’s possible that the developed modules in our intervention don’t necessarily reflect the priorities of children. Relatedly, our intervention modules were designed to support education, knowledge, and skill development in furtherance of promoting AT among children, and how specific sets of these modules may work best in complement to each other. As a consequence of this design, it is unclear how the educational content of this intervention might function in tandem with other strategies such as school promotions (e.g., ‘walk to school’ days), enforcement policies (e.g., new school speed zones), or engagement initiatives (e.g., community watch programs). Third, with respect to generalizing findings from our sample, it should be noted that majority of our participants were located in the Greater Toronto Area, a major metropolitan area in southern Ontario, therefore applying the recommendations of this study to rural or other comparatively small urban settings should be done cautiously. Given that our recruitment process was attached to a previous AST study which surveyed participants on various AT issues, this method may likely have introduced some level of selection bias (i.e., higher number of individuals who care more about AT as a general issue) into the study. Additionally, we recognize that with a sample of 57 dyads and the pilot nature of this study, results were not powered to examine intervention efficacy; however, this was an exploratory evaluation and multiple feasibility studies have been published with equivalent or smaller group sizes [87, 89]. As it pertains to the internal validity of the intervention, while we sought to address multiple AT concepts and did so through randomly allocating participants into a multi-group (i.e., intervention groups and a control group) factorial design, the intervention design—for logistical reasons—stipulated a 4–16-day timeframe for completion. Such a timeline for the intervention evaluation is relatively short when compared to seasonal variations/changes, and consequently there is the potential for some regression in knowledge retention if follow-ups were conducted further from the baseline. Thus, future studies should consider implementing longer follow-ups to evaluate the transferability of the skills and behaviours to practice. Last, this intervention was implemented and ran with what is presumed to be a sample of relatively healthy children. The transferability of our findings to specific sex/gender, physical health status, mental health condition, etc. should be done judiciously.

## Conclusion

Although promoting AT can be an efficient way through which to improve children’s health, many do not engage due to issues related to pedestrian education, knowledge, and skills. To address these concerns and support children’s AT, this manuscript presents the development and evaluation of the feasibility and effectiveness of an online road safety intervention for children and parents. Feasibility analyses suggest certain components for each module (e.g., activities, videos) should be evaluated for appropriateness with respect to the studied age group (i.e., 9–13 years) to improve engagement and retention, but program materials are likely to be scalable. Preliminary effectiveness analyses indicated that AT knowledge scores weren’t significantly improved at post-intervention; however, children’s road safety knowledge scores saw significant improvements in groups 4, 6, 7, and 8, which may be due to the presence of the ‘wheeling safety and skills’ module. Preliminary effectiveness on parental AT practices saw improvements in groups 1, 6, and 8, but a decline in the guided choice measure for group 7. Meanwhile, parents in group 4 perceived significantly less road safety barriers upon completion of the program. Similar to the child analyses, groups that featured the ‘wheeling safety and skills’ module tend to exhibit significantly higher guided choice scores upon completion of the program compared to those who did not receive this component. It is recommended that future research focus on refining the individual components of each module to better reflect the priorities of children and configure improved optimization criterion, and subsequently test these refined components among larger samples.

### Electronic supplementary material

Below is the link to the electronic supplementary material.

Supplementary Material 1

## Data Availability

The datasets generated and/or analyzed during the current study are not publicly available due research ethics board requirements, but may be available from the corresponding author on reasonable request.

## Abbreviations

AT: Active travel
AST: Active School Travel
ASRTS: Active and Safe Routes to School
ICA: Inductive content analysis
MOST: Multiphase Optimization Strategy
OEI: Online education intervention
PA: Physical activity
PAPP: Physical Activity Parenting Practices
PASTEB-C: Perceived AST Barriers and Enablers – Child
PASTEB-P: Perceived AST Barriers and Enablers – Parents
RA: Research assistant
STP: School Travel Planning

## Footnotes

1 There are important legislative differences between Canadian provinces regarding transportation regulations, thus our intervention was designed according to Ontario legislation.

2 The post-intervention survey was completed immediately after completing the program, which varied between 4 and 16 days after baseline depending on the number of modules that were assigned to participants.

3 After the study was complete, control participants, along with all others, were offered the opportunity to receive the entire intervention.

4 Module feedback included responses from children who completed the program and those who were lost to the post-intervention survey follow-up.
