# Supplementary material for: Applying the multiphase optimization strategy to evaluate the feasibility and effectiveness of an online road safety education intervention for children and parents: a pilot study
Source: BMC Public Health. 2024 Jul 4;24:1782. doi: 10.1186/s12889-024-19208-z (PMC11223427; doi:10.1186/s12889-024-19208-z)
Supplement: Supplementary file 1 — Supplementary Material 1 [file 12889_2024_19208_MOESM1_ESM.docx]

## **Additional File 1**

**Table S1**. Module topics, objectives, learning outcomes, and activity outline.

| **Lesson** | **Objectives** | **Learning Outcomes** | **Activities** |
| --- | --- | --- | --- |
| **Module 1:** Active Travel Knowledge | - To familiarize children with the definition of active transportation. - To explore the benefits of active transportation on physical, mental, and social well-being - To educate children on the effects of active transportation on air quality and the environment | After completing this lesson, children should be able to:   - Explain the different methods of active transportation. - Make connections between active transportation and climate change/environment. - Make connections between active transportation and overall health (e.g., physical health, mental health) | Online Activities   - Active Travel Story   - *Description*: Children would write a narrative story on the topic of active school travel.   - *Credits*: Elgin London Middlesex Oxford (ELMO) Active & Safe Routes to School (ASRTS) committee - Mapping your Route to School   - *Description*: Using an interactive mapping tool, children would measure how far they travel to school   - *Credits*: Toronto and Region Conservation Authority   Hands-on Activities   - Active Travel Passport   - *Description:* children would track/report their method of travelling to and from school each day   - *Credits*: ELMO ASRTS - Hop!   - *Description:* a web-app that allows users to track their steps and trips to/from school and other destinations   - *Credits*: Ottawa Student Transportation Authority and EnviroCentre |
| **Module 2:** Pedestrian Safety Skills | - To provide students with the skills to demonstrate pedestrian road safety techniques. - To encourage students to be more aware of their surrounding when walking to school or other destinations in their community - To identify safety risks associated with being a pedestrian. - To explore the best practices of exiting a school bus at an intersection. | After completing this lesson, children should be able to:   - Define “pedestrian” - Know the basic rules of crossing different types of roads safely (i.e., PXOs, crosswalk, roundabouts, mid-blocks, parking lots) - Understand their responsibilities on the road as a pedestrian. - Safely exit a school bus to cross the road at an intersection. | Online Activities   - WalkSafe Crossword Puzzle   - *Description:* This puzzle was designed to get students to familiarize themselves with the various road safety topics that were discussed in the readings and videos.   - *Credits*: Ottawa Safety Council - Take the Lead Game   - *Description:* Children would navigate an avatar through different road safety scenarios of which they must make the correct decisions to win the game   - *Credits*: Student Transportation Services of Waterloo Region   Hands-on Activities   - Road Safety Scavenger Hunt   - *Description:* Children are asked to find the items on the checklist and create a slideshow presentation of their findings   - *Credits*: Ottawa Safety Council |
| **Module 3:** Signs and Infrastructure | - To build an understanding of various traffic structures in the community (i.e., road signs, construction signs) - To teach students the concept of right of way, and how right of way applies to pedestrians and motorists at intersections | After completing this lesson, children should be able to:   - Correctly identify road signs that they may encounter on their trip to school or other local destinations in their community and know the behaviours associated with these signs:   - Stop Sign   - Yield Sign   - No Pedestrians Allowed Sign   - Railway Crossing Signs   - School Zone Sign   - Bicycle Route Sign   - No Bicycles Allowed   - Pedestrian Crossover Sign   - Do Not Enter Sign   - One Way Sign - Safely navigate through construction zones. - Determine who has right of way at different road infrastructures. | Online Activities   - Match the Traffic Signs   - *Description:* Children must match each traffic sign to their associated behaviour or definition by drawing a line to connect them   - *Credits*: Ontario Active School Transportation (OAST) & Porcupine Health Unit   Hands-on Activities   - iSpy Signs   - *Description:* This exercise teaches children to be observant and to recognize the various traffic and community signs in their neighbourhod by taking pictures/videos of the items listed on the checklist)   - *Credits*: ELMO ASRTS and Human Environments Analysis Laboratory |
| **Module 4:** Wheeling Safety and Skills | - To introduce children to the legal requirements for cycling, scooting, and rollerblading in Ontario - To teach students how to ride safely on the road and/or sidewalk. - To provide children with the resources to apply their biking skills (i.e., hands-on bike skills programs, exercises that they can do on their own). | After completing this lesson, children should be able to:   - Know what bikes and cyclists are required to have by law. - Understand how to properly fit and take care of helmets. - Know the key elements to getting their bike fitted. - Understand when and how to use the ABC check. - Know how to do shoulder checks and hand signals to safely stop and make turns on the road. - Know when it is appropriate to ride on sidewalks. - Understand the rules of the road. - Identify potential dangers on the road and on the sidewalk | Online Activities   - Drag and Drop – Cycling Edition   - *Description:* The objective of this interactive game is to categorize bike-related equipment (e.g., bell, lights, helmet, etc.) into their respective categories: “required” or “recommended”.   - *Credits:* Human Environments Analysis Laboratory - Do you know your ABCs?   - *Description:* This interactive game will get students to drag and drop each step of the ABCD bike check into the correct sequence   - *Credits:* Human Environments Analysis Laboratory   Hands-on Activities   - Bike Rodeo   - *Description:* This exercise will teach children to develop the basic bike safety and handling skills needed for safe cycling. Children would snap a photo/video of them performing different procedures   - *Credits:* Ottawa Safety Council and London Cycle Link |

**Table S2**. Frequency counts for the qualitative assessment of participant module feedback.

| **Themes** | **Subtheme** | **Description** | **Quotation Examples** | **Active Travel Knowledge** | **Pedestrian Safety and Skills** | **Signs and Infrastructure** | **Wheeling Safety and Skills** | **Total** |
| --- | --- | --- | --- | --- | --- | --- | --- | --- |
| **Cons** | | | | | | | | |
| Activities (n=48) | Difficulty | Comments about certain activities being difficult to complete | “the active map activity was difficult and frustrating” (Child; Module 1) | 8 | 5 | 1 | 0 | 14 |
|  | Duration | How long it took to complete the activities | “I don't like that some of the activities take a very long time to do” (Child; Module 2)  “Least favourite was perhaps Signs and Infrastructure because it perhaps could have been more condensed.” (Parent; Module 3) | 3 | 3 | 1 | 1 | 8 |
|  | Engagement | Comments about activity enjoyment and interactivity, and how it could be improved (e.g., adding more creative components, gamification, etc.) | “More games would make it more fun and easy” (Child; Module 2)  “Lease favourite was the scavenger hunt...The hunt was fun but not making a slideshow” (Parent; Module 2) | 2 | 2 | 0 | 2 | 6 |
|  | Technical Issues | Concerns relating to technical difficulties (e.g., couldn't load third party activity website on device, difficulty with uploading activities on OWL, etc.) | “I struggled with the mapping module and submitting of files module even with the help of my parent.” (Child; Module 1) | 3 | 1 | 2 | 0 | 6 |
|  | Convenience | How well the activity fits into the participant's schedule (includes weather-related reasons). | “My favorite part was crossword [activity]. The scavenger hunt was fun to but would be better in the summer” (Child; Module 2)  “but we didn' t have time to finish the activities because too much work” (Parent; Module 1) | 2 | 2 | 2 | 2 | 8 |
|  | Parental Support | Indication that children are heavily relying on parent's support to complete tasks (e.g., lack of independent tasks). | “There was a lot that I needed help from a parent to complete” (Child; Module 1) | 4 | 0 | 1 | 1 | 6 |
| Videos (n=15) | Length | Amount of videos and duration of videos. | “My least favourite part about it was that there was too many videos.” (Child; Module 2) | 2 | 1 | 2 | 3 | 8 |
|  | Content | Signs that there was a lack of interest in the video content and/or suggestions on how to make it more enjoyable | “Add more interesting videos. (Child; Module 3) | 1 | 2 | 1 | 0 | 4 |
|  | Accessibility | Concerns relating to the audio. | “My least favourite part was when the volume of the video's were too quiet so I could not hear them very well” (Child; Module 4) | 0 | 0 | 2 | 1 | 3 |
| Module Instructions (n=3) |  | Comments regarding instructional clarity. | “It was hard to follow the different tasks that needed to be done” (Child; Module 3) | 0 | 1 | 2 | 0 | 3 |
| Readings (n=5) |  | Concerns related to the readings’ length (e.g., too much text) and/or content (e.g., interactivity) | “My least favourite part of the module was the videos and there was also lots of reading.” (Child; Module 3) | 1 | 1 | 3 | 0 | 5 |
| Quizzes (n=7) |  | Comments regarding the overall design of the quiz (e.g., length, question clarity) and difficulty of the questions. | “IDK maybe [add] like a harder question at the end of the quiz.” (Child; Module 3) | 3 | 1 | 2 | 1 | 7 |
| User Interface (n=5) |  | Comments regarding the OWL website interface (e.g., not user-friendly, difficult navigation) | “I feel you can improve the module buy fixing a glitch where when you enter a module and do back to the page with the slides you have to reload the entire page because the slides did not load properly” (Child; Module 1) | 3 | 1 | 0 | 1 | 5 |
| **Pros** | | | | | | | | |
| Videos (n=19) |  | Comments suggesting that the video portion of the modules were their favourite part of the module(s) and/or that they were enjoyable. | “i liked the videos because they taught me some things.” (Child; Module 1) | 8 | 6 | 4 | 1 | 19 |
| Activities (n=42) |  | Comments saying that the activities were their favourite part of the module(s) and/or that they were enjoyable. | “i like the walking activity because I played it with friends when we walked home from school.” (Child; Module 1)  “My favorite module was the active travel knowledge module because I really like the Hop activitie and the Active Travel Passport.” (Parent; Module 1) | 12 | 9 | 9 | 12 | 42 |
| Quizzes (n=12) |  | Comments suggesting that the quizzes were their favourite part of the module(s) and/or that they were enjoyable. | “The quiz was fun to do. I wanted to get them all right.” (Child; Module 3) | 7 | 1 | 4 | 0 | 12 |

**Table S3**. Frequency counts for the qualitative assessment of the overall program feedback

| **Themes** | **Definition** | **Quotation Examples** | **Counts** |
| --- | --- | --- | --- |
| Increased Knowledge (n=23) | Increased understanding of the topics (e.g., benefits of active transportation, rules of the road, bike skills, etc.) | “I now know what the signs are for and what the rules are to safely use the road as a pedestrian” (Child; Group 2)  “[my child] understands the value of biking. Would like to do more on safety and signs.” (Parent; Group 6) | 23 |
| Change in Behaviour (n=11) | Involves increased road awareness, increased use of active transportation, and/or increased active transportation motivation. | “it tells me the benefits which makes me want to walk to school more” (Child; Group 1)  “I would say it has equipped us with the skills to continue to use active transportation. I will try to get them to walk even more.” (Parent; Group 5) | 11 |

**Table S4.** Sample characteristics (age, school commuting behaviour, physical activity behaviour) of the children who have completed the Online Road Safety Education program (N=57)

| **GROUP** | **Mean age (years)** | **Number of children who live within walking distance to school (%)^a^** | **Number of AST commuters at baseline**  **(%)** | **Number of AST commuters at post-intervention**  **(%)** | **Number of passive commuters at baseline (%)** | **Number of passive at post-intervention**  **(%)** | **Mean baseline PA (days)** | **Mean post-intervention PA (days)** |
| --- | --- | --- | --- | --- | --- | --- | --- | --- |
| Control | 9.8 | 4 (66.7) | 4 (66.7) | 4 (66.7) | 2(33.3) | 2 (33.3) | 5.2 (3.03) | 6.0 (2.76) |
| 1 | 10.5 | 3 (50.0) | 6 (100.0) | 6 (100.0) | 0 (0.0) | 0 (0.0) | 4.67 (1.50) | 6.33 (1.86) |
| 2 | 10.0 | 3 (50.0) | 5 (83.3) | 4 (66.7) | 1 (16.7) | 2 (33.3)) | 4.50 (0.84) | 4.67 (1.51) |
| 3 | 9.7 | 4 (66.7) | 4 (66.7) | 5 (83.3) | 2 (33.3) | 1 (16.7) | 4.40 (1.94) | 5.33 (1.96) |
| 4 | 10.3 | 4 (57.1) | 5 (71.4) | 5 (71.4) | 2 (28.6) | 2 (28.6) | 5.00 (1.73) | 5.86 (2.26) |
| 5 | 10.5 | 6 (100.0) | 5 (83.3) | 1 (16.7) | 5 (83.3) | 1 (16.7) | 3.17 (2.13) | 4.00 (2.28) |
| 6 | 10.0 | 5 (83.3) | 4 (66.7) | 2 (33.3) | 4 (66.7) | 2 (33.3) | 4.00 (1.89) | 6.33 (1.86) |
| 7 | 9.4 | 5 (71.4) | 4 (57.1) | 6 (85.7) | 3 (42.9) | 1 (14.3) | 5.29 (1.38) | 6.14 (1.34) |
| 8 | 10.3 | 3 (42.9) | 2 (28.6) | 5 (71.4) | 2 (28.6) | 5 (71.4) | 6.14 (0.90) | 5.86 (2.27) |
| ^a^ Parent self-reported whether they believe that the child lives within walking distance to school  Acronyms: AST – Active School Travel; PA – Physical Activity | | | | | | | | |

**Table S5.** A Description of the parental outcome measures

| **Parental AT Practices (Adaptation from the Physical Activity Parenting Practices Item Bank [PAPP])** | |
| --- | --- |
| **Measures** | **Description** |
| Guided Choice | A component of larger autonomy promotion and refers to approaches that parents employ to support child independence (e.g., ‘Allow your child(ren) to choose the physical activity/sports you do as a family (whether you go for a walk, hike, bike ride, or play an active game)’). |
| Non-directive Support | The value and encouragement of PA through the strategies of co-participation, modeling, and monitoring (e.g., ‘Participate in any physical activity (incl. active school travel) with your child(ren)’). |
| Supportive Expectation | Similar to non-directive support, refers to and captures parental encouragement for PA through their personal beliefs and expectations about various topics (e.g., ‘Believe that your child(ren) should participate in some form of physical activity or sports on most days of the week’). |
| Autonomy Support | Autonomy support involves more general promotion of individual allowance to decide on activity engagement (e.g., ‘Praise your child(ren) for being physically active or for participating in sports or physical activity classes’). |
| **Parental Controls and Perceived AT Barriers (Adapted from the Perceived Active School Travel Enablers and Barriers – Parents [PASTEB-P] Questionnaire)** | |
| **Measure** | **Description** |
| Parental Controls | This measure delineates the extent to which parents afford their children independent mobility (specifically AST) opportunities. This measure was examined through asking participants about a series of travel situations and if they allowed their child to engage in independent travel. Overall, 13 items were used: walk to/from school with adults, peers, alone; bike to/from school with adults, peers, alone; roll to/from school with adults, peers, alone; cross busy roads alone at crosswalks; travel on public transportation alone; travel to non-school destinations in the neighbourhood; and go out alone after dark. Items were recorded as dichotomous responses (e.g., Yes, No). |
| Perceived AT barriers | This measure captured a variety of different constructs including convenience (e.g., “We do not have enough time in the morning”), road safety (e.g., “A child must cross busy roads”), and social safety (e.g., “Unsafe because of strangers in the neighbourhood. For the purpose of this study, we have examined only the ‘road safety’ construct. |
| **Parental AT Practices (Adaptation from the Physical Activity Parenting Practices Item Bank [PAPP])** | |
| **Measures** | **Description** |
| Guided Choice | A component of larger autonomy promotion and refers to approaches that parents employ to support child independence (e.g., ‘Allow your child(ren) to choose the physical activity/sports you do as a family (whether you go for a walk, hike, bike ride, or play an active game)’). |
| Non-directive Support | The value and encouragement of PA through the strategies of co-participation, modeling, and monitoring (e.g., ‘Participate in any physical activity (incl. active school travel) with your child(ren)’). |
| Supportive Expectation | Similar to non-directive support, refers to and captures parental encouragement for PA through their personal beliefs and expectations about various topics (e.g., ‘Believe that your child(ren) should participate in some form of physical activity or sports on most days of the week’). |
| Autonomy Support | Autonomy support involves more general promotion of individual allowance to decide on activity engagement (e.g., ‘Praise your child(ren) for being physically active or for participating in sports or physical activity classes’). |
| **Parental Controls and Perceived AT Barriers (Adapted from the Perceived Active School Travel Enablers and Barriers – Parents [PASTEB-P] Questionnaire)** | |
| **Measure** | **Description** |
| Parental Controls | This measure delineates the extent to which parents afford their children independent mobility (specifically AST) opportunities. This measure was examined through asking participants about a series of travel situations and if they allowed their child to engage in independent travel. Overall, 13 items were used: walk to/from school with adults, peers, alone; bike to/from school with adults, peers, alone; roll to/from school with adults, peers, alone; cross busy roads alone at crosswalks; travel on public transportation alone; travel to non-school destinations in the neighbourhood; and go out alone after dark. Items were recorded as dichotomous responses (e.g., Yes, No). |
| Perceived AT barriers | This measure captured a variety of different constructs including convenience (e.g., “We do not have enough time in the morning”), road safety (e.g., “A child must cross busy roads”), and social safety (e.g., “Unsafe because of strangers in the neighbourhood. For the purpose of this study, we have examined only the ‘road safety’ construct. |
